# Temperature-Dependent Expression of *NodC* and Community Structure of Soybean-Nodulating Bradyrhizobia

**DOI:** 10.1264/jsme2.ME15114

**Published:** 2016-02-13

**Authors:** Sokichi Shiro, Chika Kuranaga, Akihiro Yamamoto, Reiko Sameshima-Saito, Yuichi Saeki

**Affiliations:** 1 Faculty of Life and Environmental Science, Shimane University1060 Nishikawatsu, Matsue, Shimane 690–8504Japan; 2 Faculty of Agriculture, University of Miyazaki1–1 Gakuenkibanadai-Nishi, Miyazaki, Miyazaki 889–2192Japan; 3 Faculty of Agriculture, Shizuoka University836 Otani, Suruga-ku, Shizuoka, Shizuoka 422–8529Japan

**Keywords:** bradyrhizobia, *nodC* gene, temperature, community structure

## Abstract

In order to assess the physiological responses of bradyrhizobia and competition for the nodulation of soybean at different temperatures, we investigated the expression of the *nodC* gene at 20, 25, and 30°C and the abilities of bacteria to nodulate soybean in microcosms at day/night cultivation temperatures of 23/18°C, 28/23°C, and 33/28°C for 16/8 h. We tested five *Bradyrhizobium* USDA strains: *B. diazoefficiens* USDA 110^T^ and 122, *B. japonicum* USDA 123, and *B. elkanii* USDA 31 and 76^T^. The expression of *nodC* was up-regulated by increasing culture temperatures in USDA 110^T^, 122, 31, and 76^T^, but was down-regulated in USDA 123. The proportions of USDA 110^T^ and 122 within the community were the greatest at 28/23°C. The population of USDA 31 increased, whereas that of USDA 123 decreased with increasing cultivation temperatures. On the other hand, infection by USDA 76^T^ was not detected, and low numbers of USDA 76^T^ nodules confirmed its poor nodulation ability. These results indicate that the competitiveness of and infection by USDA 110^T^, 122, 123, and 31 for soybean nodulation depend on cultivation temperatures, and suggest that the temperature dependence of *nodC* expression affects the bradyrhizobial community structure.

Soybean (*Glycine max* [L.] Merr.) is an important crop plant that forms root nodules by infections with rhizobia, which fix atmospheric nitrogen as ammonia through these nodules. *Bradyrhizobium diazoefficiens*, *B. japonicum*, and *B. elkanii* are major soybean-nodulating rhizobia (8, 16, 20). The inoculation of soybean with bradyrhizobia may improve nitrogen fixation, resulting in increased soybean yield. However, the efficiency of the inoculum may be poor if it cannot compete with indigenous bradyrhizobia in the soil or is unable to establish an efficient symbiosis with the host plant due to low compatibility (42). In order to overcome this issue, a clearer understanding of the ecology of indigenous soybean-nodulating rhizobia is needed in terms of their genetic diversity, geographical distribution, compatibility with soybean, and environmental factors associated with the localization and dominance of strains in soil.

Saeki *et al.* (32) investigated the genetic diversity and geographical distribution of indigenous soybean-nodulating bradyrhizobia collected from five sites in Japan (Hokkaido, Fukushima, Kyoto, Miyazaki, and Okinawa) by analyzing PCR restriction fragment length polymorphisms (RFLP) of the 16S-23S rRNA gene internal transcribed spacer (ITS) region. The distribution of bradyrhizobia strongly correlated with latitude: representative clusters changed from north to south in the order of *B. japonicum* strains USDA 123, 110 (*B. diazoefficiens* USDA 110^T^), 6^T^, and *B. elkanii* strain USDA 76^T^ (33, 35, 36). These findings suggested that environmental factors such as temperature influenced the localization of Japanese indigenous bradyrhizobia. Saeki *et al.* (34) investigated the dominance of three *B. japonicum* strains and one *B. elkanii* strain at different temperatures in soil and liquid media, and suggested that temperature affected the occupancy of indigenous bradyrhizobia in soil. Adhikari *et al.* (1) revealed the genomic diversity of soybean-nodulating bradyrhizobia in relation to climate, as determined by altitude, and to soil properties, such as pH, in Nepal. Suzuki *et al.* (43) also reported the prominent effects of temperature on competition between *B. japonicum* and *B. elkanii* strains that corresponded with the distribution of bradyrhizobial species in Nepal. In USA, the world’s biggest soybean producer, soybean is grown at similar latitudes to those in Japan. Shiro *et al.* (39) investigated the relationship between the genetic diversity of indigenous soybean-nodulating bradyrhizobia and their geographical distribution in USA using nine soil isolates from eight states: as in Japan, the major clusters changed from *B. japonicum* USDA 123 in the northern states to *B. elkanii* in the central and southern states. The indigenous American bradyrhizobial community structure also strongly correlated with latitude. These results suggest a relationship between the geographic distribution of indigenous soybean-nodulating rhizobia and soil temperature (and its variations due to latitude and altitude) as well as soil pH. Shiro *et al.* (38) investigated the nodulation tendencies and community structures of indigenous bradyrhizobia on soybean cultivars with different *Rj* (nodulation regulatory gene) genotypes at day/night culture temperatures of 33/28°C, 28/23°C, and 23/18°C for 16/8 h; the findings obtained suggested that the *Rj* genotype and culture temperature affected the nodulation tendencies and community structures of bradyrhizobia. These findings indicate that changes in bradyrhizobial community structures induced by temperature are caused by differences in the responses of symbiosis-related genes such as nodulation (*nod*) genes.

In order to test this hypothesis, we investigated the temperature-dependent responses of the *nodC* gene, which encodes NodC, the first enzyme in the biosynthesis pathway of Nod factor using the substrate UDP-*N*-acetyl glucosamine (13, 18), and competition for nodulation at different temperatures, with the aim of determining whether the temperature dependency of the expression of the *nodC* gene contributes to infection by bradyrhizobia for soybean nodulation.

## Materials and Methods

### Bradyrhizobial strains and culture conditions

*B. diazoefficiens* strains USDA 110^T^ and 122, *B. japonicum* strain USDA 123, and *B. elkanii* strains USDA 31 and 76^T^ were used in the present study. In a quantitative real-time PCR (qPCR) analysis, they were grown in HEPES-MES (HM) broth medium (7) supplemented with 0.1% L-arabinose (37). In order to estimate nodulation abilities, these strains were grown in yeast extract-mannitol broth (YMB) medium (45).

### RNA extraction, cDNA synthesis, and qPCR analysis

Regarding RNA extraction, bacteria were pre-cultured at 28°C in 50 mL of HM broth medium for 3 d and then scaled up to 200 mL with the addition of fresh HM broth medium. The bacteria culture was further conducted at 28°C for log-phase growth (OD_600_ = 0.3–0.5). Cell culture aliquots were diluted to 200 mL with fresh HM broth medium to OD_600_ = 0.1, and genistein (Nacalai Tesque, Kyoto, Japan) was added to a final concentration of 5 μM in order to induce the expression of *nodC* (10, 22, 29, 46, 47, 50). These cultures were grown at 20, 25, or 30°C for 24 h, and cells were then immediately harvested by centrifugation and lyophilized. Total RNA was extracted with ISOGEN-LS (Nippon Gene, Tokyo, Japan) according to the manufacturer’s instructions. cDNA was synthesized with the PrimeScript^®^ RT Reagent Kit with gDNA Eraser (Perfect Real Time) (TaKaRa Bio, Shiga, Japan) according to the manufacturer’s instructions. qPCR was performed with SYBR^®^ *Premix Ex Taq*^TM^ II (Tli RNaseH Plus) (TaKaRa Bio) in the Thermal Cycler Dice^®^ Real Time System TP800 (TaKaRa Bio), and the relative expression quantity of *nodC* was calculated from TP800 data. The specific primers for *nodC* and control genes for the qPCR analysis are shown in Table 1. The expression levels of three biological replicates in each treatment were normalized to those of the *sigA* gene. The *sigA* gene, which encodes a primary sigma factor, is used as a housekeeping control gene because its expression is independent of temperature (28, 44, 46, 47). Relative gene expression among the treatment groups was quantified using the 2^−ΔΔC^_T_ method (21, 48). A real-time PCR analysis was conducted at three replications.

### Nodulation ability and competition studies using microcosms

In order to estimate nodulation by and competition among bradyrhizobial strains, we performed experiments using soil microcosms and three soybean cultivars (Akishirome, Bragg, and Orihime; non-*Rj* genotype, 24). Bradyrhizobia were cultured in YMB medium at 28°C for 6 d, and then mixed in combinations of three strains into sterile soil (Andosol, pH [H_2_O] = 6.46, pH [KCl] = 5.22, EC = 0.03 dS m^−1^, CEC = 31.2 cmol kg^−1^) at a bacterial density of 10^6^ cells g^−1^ dry soil. Four combinations were prepared: A (USDA 31, 110^T^, and 123), B (USDA 31, 122, and 123), C (USDA 76^T^, 110^T^, and 123), and D (USDA 76^T^, 122, and 123).

In order to isolate the strains from the microcosms, we grew soybeans in 1-L culture pots. The pots were first filled with vermiculite with N-free nutrient solution (30) at 40% (v/v) water content and then autoclaved at 121°C for 20 min. Soybean seeds were surface-sterilized in 70% ethanol for 30 s followed by dilute sodium hypochlorite (0.25% available chlorine) for 3 min, and then washed in sterile distilled water. Microcosm soil (2 to 3 g) was placed in vermiculite at a depth of 2 to 3 cm, and seeds were sown on it. Plants were grown for 4 weeks in a growth chamber at one of the three temperature regimes—low (day/night, 23/18°C for 16/8 h), middle (28/23°C), and high (33/28°C)—with a weekly supply of sterile distilled water. After 4 weeks, 24 nodules were randomly collected and sterilized in 70% ethanol for 3 min followed by dilute sodium hypochlorite for 30 min, and then washed in sterile distilled water. As a negative control, it was confirmed that soybean plants grown without soil, eliminating the possibility of contamination with soybean-nodulating bacteria, formed no nodules.

Soybean-nodulating bradyrhizobia were identified by PCR-RFLP of the 16S-23S rRNA gene ITS region. Total DNA was directly extracted from nodules as described previously (15) with slight modifications (24). Each nodule was homogenized in 50 μL of BL buffer (40 mM Tris-HCl, 1% Tween 20, 0.5% Nonidet P-40, 1 mM EDTA, pH 8.0), 40 μL of sterile distilled water, and 10 μL of proteinase K (1 mg mL^−1^) and then incubated at 60°C for 20 min and 95°C for 5 min. After centrifugation, the supernatant was collected and used as the PCR template. PCR was performed using *TaKaRa Ex Taq*^®^ (TaKaRa Bio). Regarding 16S-23S rRNA gene ITS region amplification, we used the primer set BraITS-F (5′-GACTGGGGT GAAGTCGTAAC-3′) and BraITS-R (5′-ACGTCCTTCATCGCC TC-3′) (31). The PCR cycle consisted of an initial 94°C for 5 min; 30 cycles of 94°C for 30 s, 55°C for 30 s, and 72°C for 1 min; and a final 72°C for 10 min. In the RFLP analysis of the ITS region, the PCR product was digested with *Msp*I (TaKaRa Bio) at 37°C for 16 h (31). Fragments were separated by electrophoresis using 3% agarose gel and visualized with ethidium bromide.

### Inoculation test for estimation of nodulation ability

In order to estimate the nodulation ability of each bradyrhizobial strain with soybean, we inoculated each strain into each of the three soybean cultivars (Akishirome, Bragg, and Orihime). These strains were cultured in YMB medium as described above. The cultures were diluted with sterile distilled water to 10^6^ cells mL^−1^. Soybean seeds were sown into 1-L culture pots as described above without soil and inoculated with 1 mL of diluted bacterial culture per seed. Soybean plants were grown for 3 weeks in a growth chamber (28/23°C for 16/8 h) with a weekly supply of sterile distilled water. After 3 weeks, the nodules were counted. As a negative control, it was confirmed that soybean plants grown without inoculation formed no nodules.

## Results

### *nodC* expression levels at different temperatures

**Fig. 1:**
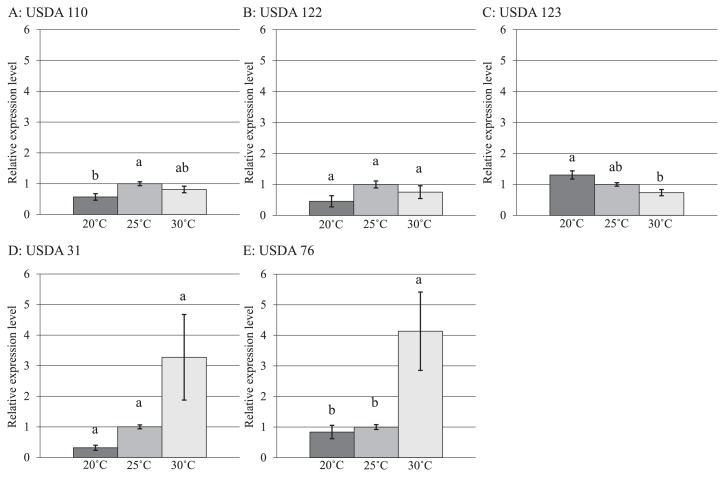
Relative expression of *nodC* in five bradyrhizobial strains. Values shown represent mean from three replications ± SE (*n*=3). Bars with the same superscript letters are not significantly different (Tukey HSD test) at *P* < 0.05.

*nodC* gene expression levels at different temperatures were estimated using the *sigA* gene as a reference gene because the expression of the 16S rRNA gene exhibits instability with changing temperatures (14). The results obtained revealed different *nodC* expression levels in each strain at low (20°C), middle (25°C), and high (30°C) temperatures (Fig. 1). *nodC* expression levels in *B. diazoefficiens* USDA 110^T^ were significantly higher at the middle temperature than at the low temperature (Fig. 1A). *nodC* expression levels in *B. diazoefficiens* USDA 122 were slightly higher at the middle temperature than at the low temperature (Fig. 1B). *nodC* expression levels in USDA 110^T^ and 122 were higher at the middle temperature than at the high temperature (Fig. 1A, B), indicating that the expression of the *nodC* gene has an optimum temperature in the vicinity of the middle to high temperatures. *nodC* expression levels in *B. japonicum* USDA 123 were significantly lower at the high temperature than at the low temperature (Fig. 1C). *nodC* expression levels in *B. elkanii* USDA 31 were slightly higher at the high temperature than at the low and middle temperatures (Fig. 1D). *nodC* expression levels in *B. elkanii* USDA 76^T^ were significantly higher at the high temperature than at the low and middle temperatures (Fig. 1E). The increased expression levels observed in USDA 31 and 76^T^ were precipitous (Fig. 1D, E).

### Change in nodulation occupancy at different cultivation temperatures

**Fig. 2:**
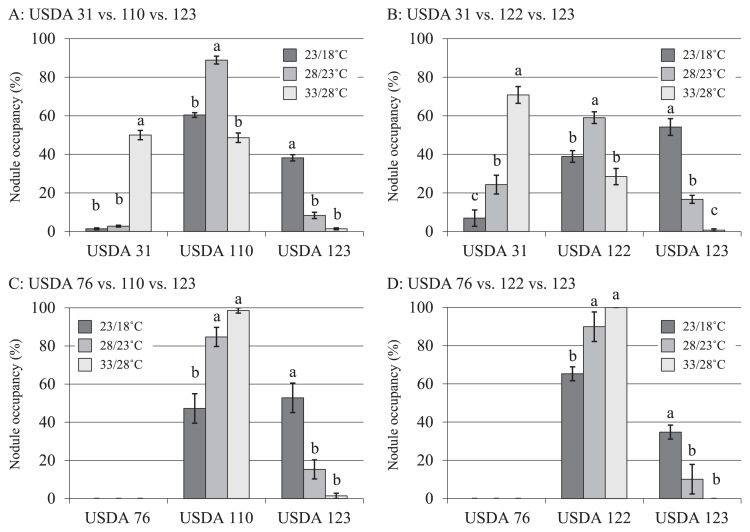
Rate of nodule occupancy by each test strain isolated from soil microcosm experiments using the indicated mixes of three strains. Values shown represent mean from three soybean cultivars, Akishirome, Bragg, and Orihime ± SE (*n*=3). Bars with the same superscript letters are not significantly different (Tukey HSD test) at *P* < 0.05.

In order to estimate the nodulation rates and competitiveness of strains, we determined the proportion of nodules infected by each strain in three-strain microcosm experiments using a PCR-RFLP analysis of the 16S-23S rRNA gene ITS region. In both combinations in which USDA 31 was present, the nodule occupancy rate of USDA 31 increased significantly at higher cultivation temperatures (Fig. 2A, B). In all four combinations in which USDA 123 was present, the occupancy rate of USDA 123 decreased significantly with increasing cultivation temperatures (Fig. 2A–D). In the presence of USDA 31 and 123, the occupancy rates of USDA 110^T^ and 122 were higher at 28/23°C than at other temperatures (Fig. 2A, B). On the other hand, unlike USDA 31, USDA 76^T^ was not detected in any combination of the nodulation occupancy test (Fig. 2C, D). In association with non-nodulation by USDA 76^T^, the occupancy rates of USDA 110^T^ and 122 increased at higher cultivation temperatures (Fig. 2C, D).

### Nodulation abilities of bradyrhizobial strains

**Fig. 3:**
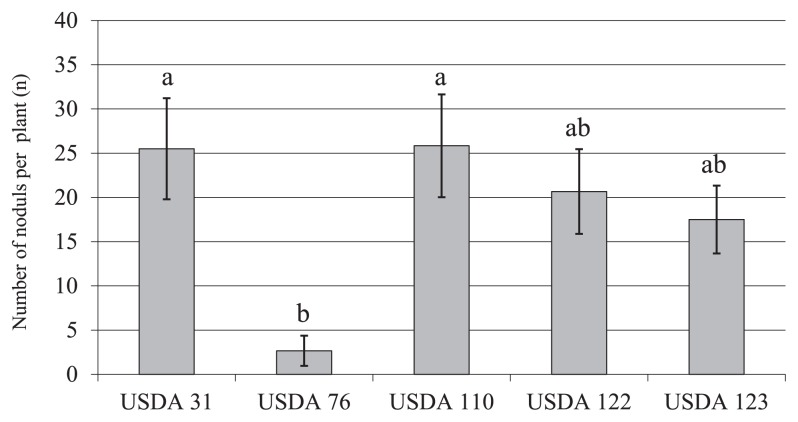
Number of nodules of each test strain on non-*Rj* genotype soybean. Values shown represent mean from total plant number of six ± SE. Three soybean cultivars, Akishirome, Bragg, and Orihime, were used each two individual. Bars with the same superscript letters are not significantly different (Tukey HSD test) at *P* < 0.05.

The nodulation ability of each bradyrhizobial strain under non-competitive conditions was shown in Fig. 3. The bradyrhizobial strain that indicated the highest nodulation ability for soybean was *B. diazoefficiens* USDA 110^T^. Sub-sequently, the order corresponding to *B. elkanii* USDA 31, *B. diazoefficiens* USDA 122, and *B. japonicum* USDA 123 indicated high nodulation ability for soybean. The nodulation ability of *B. elkanii* USDA 76^T^ had the lowest value and was significantly different from those of USDA 31 and USDA 110^T^ (Fig. 3).

## Discussion

The expression level of each *nodC* gene in the individual strain at each temperature was almost the same when corrected against the *sigA* gene (Fig. 1). Therefore, *nodC* genes in the five strains showed strain-specific temperature-dependent changes under our experimental conditions. Although significant differences were not detected among different temperatures in each strain of *B. diazoefficiens* USDA 122 and *B. elkanii* USDA 31, the patterns of *nodC* expression were similar to those of USDA 110^T^ and USDA 76^T^, for which significant differences were detected, respectively. A significant difference may have been detected in each strain of *B. diazoefficiens* USDA 122 and *B. elkanii* USDA 31 by increasing the number of replications. The production of Nod factor (a lipo-chitooligosaccharide), which triggers leguminous plant responses including the initiation of cell division to form nitrogen-fixing root nodules (9, 12), in *B. japonicum* has been shown to increase at higher rhizosphere temperatures and genistein concentrations (54). The increased production of Nod factor at higher temperatures suggests the up-regulated expression of the *nod* gene. Begum *et al.* (5) investigated the effects of incubation temperatures at 15 and 28°C on the expression of *nodC* in *Rhizobium leguminosarum*: an incubation temperature of 28°C induced the maximum expression of *nodC*, while a low incubation temperature reduced its expression. These findings appear to support our results. However, the expression of *nodC* in USDA 123 decreased from low to high temperatures (Fig. 1C). Soybean-nodulating bradyrhizobia with a similar ITS RFLP type to USDA 123 are tolerant to lower temperatures and, thus, maintain dominance at lower temperatures (32, 34, 39).

In the nodulation ability and competition studies performed using microcosms, USDA 110^T^ and 122 were dominant at 28/23°C (Fig. 2). The occupancy of USDA 31 increased, while that of USDA 123 decreased at higher cultivation temperatures (Fig. 2). USDA 123 may be able to maintain its infectious ability to soybean even under low temperature conditions because the expression of *nodC* or reverse temperature-dependent expression of *nod* genes in this strain is less sensitive to temperature (Fig. 1C). These results suggest that USDA 123 nodulates soybean more effectively than other strains under low temperature conditions. However, although USDA 76^T^ indicated the temperature-dependent expression of the *nodC* gene, nodules infected by USDA 76^T^ were not detected (Fig. 2C, D) possibly because of its low compatibility with or low ability of nodulation on soybean (Fig. 3) despite its *nodC* expression ability by genistein (Fig. 1E). Therefore, the low competitiveness of USDA 76^T^ for nodulation may have allowed for the increased occupancy of USDA 110^T^ and 122 at higher cultivation temperatures. Since the changes observed in the temperature-dependent expression of *nodC* in USDA 31, 110^T^, 122, and 123 were generally consistent with those in nodule occupancy, these results suggest that *nodC* expression levels affect the nodulation competitiveness of bradyrhizobia. Yokoyama (50) demonstrated that the expression of *nod* genes in *B. japonicum* USDA 110 (*B. diazoefficiens* USDA 110^T^), *B. elkanii* USDA 76^T^, and *Bradyrhizobium* sp. TARC 64 (isolated from soil in Thailand; 49) depended on incubation temperatures in the range of 20 to 40°C, and suggested that the transcriptional responses of the *nod* genes of USDA 110 and USDA 76^T^ were distinctly different at 23 to 35°C. Additionally, this study assessed the abilities of various bradyrhizobia (*B. japonicum* USDA 110, 122 (*B. diazoefficiens* USDA 110^T^ and 122), 123, 5033; *B. elkanii* USDA 31, 76^T^; *Bradyrhizobium* sp. TARC 64) to nodulate soybean under different temperature conditions (23/18°C, 25/25°C, and 34/28°C), and suggested that *B. japonicum* strains prefer 23/18°C and 25/25°C, while *B. elkanii* strains prefer 34/28°C. Banfalvi *et al.* (4) reported that genistein and soybean seed extract more strongly promoted the expression of *nodY* and *nodC* in *B. japonicum* USDA 110 (*B. diazoefficiens* USDA 110^T^) than daidzein. On the other hand, Kosslak *et al.* (19) investigated the expression of *nodABC* genes in *B. japonicum* strains including *B. japonicum* USDA 110 (*B. diazoefficiens* USDA 110^T^) and USDA 123 using isoflavones and soybean root extract, and reported that expression was more strongly induced by daidzein than by genistein. These findings suggest that the induction and expression of *nod* genes differ with the types of isoflavones secreted from soybean roots and also with strain. Furthermore, a decrease in the rhizosphere temperature was shown to delay the infection of soybean roots by bradyrhizobia, reduce the secretion of genistein from roots, and suppress the expression of *nod* genes (51, 52, 53). However, it increased the secretion of daidzein (27). In our study, the occupancy of *B. japonicum* USDA 123 increased as temperature decreased (Fig. 2). In addition to affecting the strength of *nod* gene expression in USDA 123, lower temperatures might also alter the types and quantities of isoflavones secreted from soybean roots.

Our results suggest that temperature is widely involved in community structure, indigenization, and dominance associated with the expression of *nod* genes and nodulation abilities of *B. diazoefficiens*, *B. japonicum*, and *B. elkanii*. Furthermore, the nodulation of four out of the five stains tested was temperature dependent, and, thus, the effect of temperature on the expression of *nodC* is an important factor affecting the nodulation of soybean and the formation of a bradyrhizobial community structure. Recent research indicates that the expression of type III secretion system (T3SS) genes in *B. japonicum* USDA 110 (*B. diazoefficiens* USDA 110^T^) is induced by soybean seed extract and genistein, and suggests that nodulation genes, especially the *nolA* and *nodD2* genes, and T3SS genes play a role in nodulation (47). Therefore, the nodulation of bradyrhizobia may be associated with several factors other than the *nodC* gene, such as salt and water-deficit stress, the existence of other rhizosphere bacteria, and protein secretion systems (2, 3, 17, 25, 26). Our results also suggest that the expression of *nodC* in USDA 76^T^ is independent of its compatibility and infection ability. Causatively, the low compatibility of *B. elkanii* USDA 76^T^ with soybean may be due to a low capacity to produce effective amounts of Nod factor to induce nodulation, which may be due, in turn, to a dysfunction in the *nod*, *noe*, and *nol* genes that function downstream of *nodC* in the biosynthesis and modification of Nod factor, or of *nodIJ* genes encoding the ABC family transporters that are involved in the secretion of Nod factor and present in all rhizobia (6, 11, 23, 40, 41). Thus, further studies on infection and compatibility with soybean are needed in order to elucidate bradyrhizobial ecology for nodulation in more detail.

## Figures and Tables

**Table 1:** Primer information used in an expression analysis of the *nodC* gene

Gene	Sequence (5′→3′)		PCR product size (bp)

	Forward primer	Reverse primer	
*sigA*	ACATGGGCATCAACGTCACC	TCGTTGTCGGTCTCGTCCTC	84
*nodC* for *B. diazoefficiens* *B. japonicum*	CGAGCGATCCGAGATTCAG	ACGTCGGCAGCAAGTATCG	135
*nodC* for *B. elkanii*	TGGACGGTGCTGACGATTG	TGTGAAGCGAGAAGCCGAG	96
